# A Case of Essential Thrombocythemia Treated for Epileptic Peri-Ictal Psychiatric Symptoms Resulting in Appropriate Palliative Care

**DOI:** 10.7759/cureus.76710

**Published:** 2025-01-01

**Authors:** Tetsuro Ishida

**Affiliations:** 1 Department of Psychiatry, Shiroishi Tomo Mental Clinic, Sapporo, JPN; 2 Department of Neuropsychiatry, School of Medicine, Sapporo Medical University, Sapporo, JPN

**Keywords:** epilepsy, epileptic peri-ictal psychiatric symptoms, essential thrombocythemia, lacosamide, liaison consultation psychiatry, palliative care

## Abstract

Myeloproliferative neoplasms are diseases characterized by excessive proliferation of myeloid cells in the bone marrow. Essential thrombocythemia (ET) is a myeloproliferative neoplasm that causes platelet proliferation. Although the prognosis for ET has improved because of cytoreductive therapies, some patients can still experience complications including bone marrow fibrosis or acquired von Willebrand disease. A 79-year-old man with a history of hyperthrombocytosis was admitted for rehabilitation after prolonged hospitalization. His treatment included hydroxyurea, which was discontinued because of side effects, and anagrelide, which was unsuccessful in controlling his platelet count. The patient showed generalized convulsions and psychiatric symptoms such as irritability. Based on his clinical symptoms and electroencephalography, he was diagnosed with peri-seizure psychiatric symptoms. Lacosamide was administered to control the seizures. He eventually died of hemorrhagic shock because of ET. However, appropriate epilepsy care improved his psychiatric symptoms and quality of life. The patient was classified as having ET with a high risk of both thrombosis and a poor prognosis because of his age, history of thrombosis, elevated white blood cell count, and JAK2 mutation. In epilepsy care, both convulsive seizures and psychiatric symptoms are important therapeutic targets. Lacosamide, which can be administered orally or intravenously and has a low risk of side effects, was chosen for the present patient. There have been no reports of epileptic seizures associated with ET in palliative medicine. Therefore, this case report is novel and useful for psychiatrists engaged in palliative care medicine and liaison consultation psychiatry.

## Introduction

Essential thrombocythemia (ET) is a myeloproliferative neoplasm characterized by the overproduction of platelets, which leads to an increased risk of thrombosis, bleeding, and progression to myelofibrosis [1,2]. Historically, the prognosis for patients with ET was poor, but advances in cytoreductive therapy have resulted in a life expectancy for patients with ET that is now almost normal. However, complications such as myelofibrosis and acquired von Willebrand disease remain important [2]. In elderly patients with psychiatric symptoms, epilepsy is often not diagnosed. In this population, the seizure recurrence rate is high (66% to 90%), and early initiation of antiepileptic drugs is necessary [3,4]. New antiepileptic drugs such as lacosamide, perampanel, levetiracetam, and brivaracetam provide new treatment options, but the most suitable drug for elderly patients remains unclear [5,6]. Here, we present a case of ET in which the management of epilepsy with lacosamide improved psychiatric symptoms and quality of life, ultimately supporting the goals of palliative care. This case highlights the importance of addressing both neurological and psychiatric symptoms in the palliative care of patients with ET.

## Case presentation

A 79-year-old male presented with pneumonia, heart failure, and splenic infarction associated with disseminated intravascular coagulation syndrome. On admission, hydroxyurea was discontinued and treatment with antibiotics, diuretics, and prednisone (30 mg/day) was initiated, which reduced the severity of his lower-limb ulcers, pneumonia, and heart failure. Then, the prednisone dose was tapered to 5 mg/day. During this period, the patient had accurate orientation and no psychiatric symptoms. However, his independence in daily living decreased because of the loss of strength associated with prolonged bed rest. Thus, he was indicated for rehabilitation.

The patient had no specific family history of hematological disease. At admission, his height was 167 cm, weight was 58.3 kg, temperature was 37.3°C, blood pressure was 132/91 mmHg, pulse was 97 beats/min, and percutaneous oxygen saturation was 95% (room air). He had a clear consciousness state, with no psychotic symptoms such as hallucinations or delusions, and no mood symptoms such as irritability or depression. He showed generalized muscle weakness of approximately 4/5 on the manual muscle test, which was likely caused by prolonged hospitalization. However, there were no other abnormal neurological findings such as paralysis or convulsions.

On admission, blood tests showed high white blood cell (26,580 cells/μL) and platelet (190.0×10^4^ cells/μL) counts and an HgbA1C value of 11.5. There were also abnormalities of the coagulation and fibrinolysis system, with a prothrombin time of 23.1 sec, activated partial thromboplastin time of 69.6 sec, fibrinogen quantity of 229 mg/dL, and D-dimer level of 1.7 μg/mL. Genetic testing showed mutations in the JAK gene. Chest computed tomography showed bilateral pleural effusions and a pale peri-bronchial frosted shadow and cardiac enlargement in the right upper lobe. The spleen showed scattered areas of post-infarction hypo-absorption. Elevated bone marrow density associated with hematological disease was also observed. Upper gastrointestinal endoscopy showed a hiatal herniation of the esophagus but no bleeding or ulceration. Pathological examination of his bone marrow showed age-inappropriate marked hyperplastic marrow. Gitter staining also showed increased reticular fiber growth, which was suggestive of myeloproliferative neoplasm.

Because the patient had a prior adverse reaction to hydroxyurea, we initiated treatment with the cytostatic agent anagrelide (1.0 mg/day) and the antiplatelet agent aspirin (100 mg/day). Additionally, feburic (10 mg) was prescribed for hyperuricemia, a side effect of cytostatic therapy, and polaprezinc (150 mg), a peptic ulcer agent, was prescribed for gastritis.

**Figure 1:**
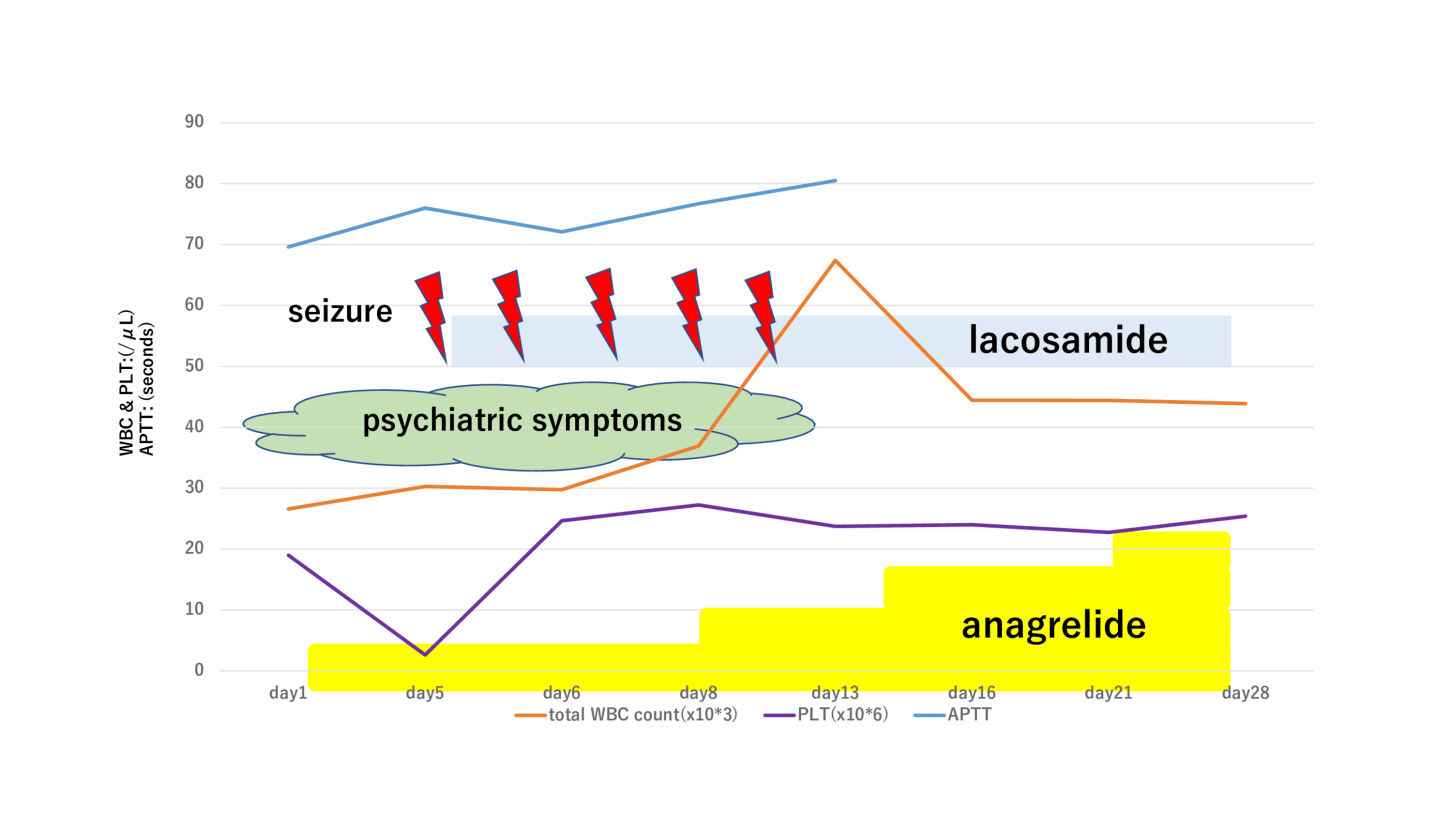
Seizures, psychiatric symptoms, and the effect of lacosamide. Increasing anagrelide was not effective in treating hyperthrombocythemia. Lacosamide suppressed the psychiatric symptoms and seizures. WBC: white blood cell; PLT: platelet count; APTT: activated partial thromboplastin time

Detailed dosage and blood test results during hospitalization are shown in Figure 1 and Table 1. Because the patient was at high risk of thrombosis (e.g., stroke or myocardial infarction), gentle rehabilitation was first started at the bedside. On day 2, the patient suddenly showed signs of irritability. He claimed that the food was not tasty and that such gentle rehabilitation was not sufficient. The hematologist provided careful informed consent again. The psychiatrist (author) provided psychotherapy, which mainly involved counseling. Nurses and rehabilitation therapists also tried to listen to him fully and show understanding. However, the patient’s verbal abuse of the medical staff continued.

**Table 1:** Laboratory data. WBC: white blood cell; PLT: platelet count; APTT: activated partial thromboplastin time; HGB: hemoglobin; PT: prothrombin time; PT-INR: prothrombin time international normalized ratio; BNP: B-type natriuretic peptide; CRP: C-reactive protein

Day	Total WBC count (x10^3^)	HGB (g/dL)	PLT (x10^6^)	APTT	PT (seconds)	D-dimer (μg/mL)	PT-INR	BNP (pg/mL)	CRP (mg/dL)
Day 1	26.58	11.5	19	69.6	23.1	1.7	2.11	86.8	5.17
Day 5	30.28	12	2.62	76	21	1.6	1.92	-	-
Day 6	29.74	11.6	24.65	72.1	19.9	1.6	1.82	-	-
Day 8	36.93	12.3	27.25	76.7	23	1.5	2.11	-	-
Day 13	67.39	12.8	23.73	80.5	24.2	1.5	2.22	-	-
Day 16	44.44	13	24	-	-	-	-	-	-
Day 21	44.41	12.8	22.75	-	-	-	-	-	-
Day 28	43.88	12	25.4	-	-	-	-	-	-
Reference ranges	3.30-8.60	13.7-16.8	0.158-0.348	23-40	9.0-13.0	-1	0.80-1.20	-18.4	-0.14

**Figure 2:**
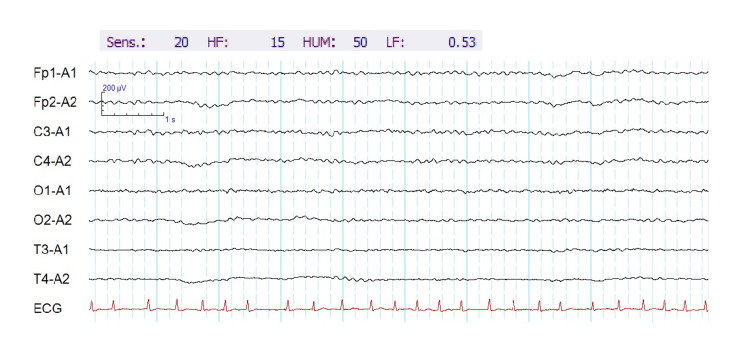
Unloaded electroencephalography (EEG) with eyes closed. The patient’s unloaded EEG results showed no obvious abnormalities.

**Figure 3:**
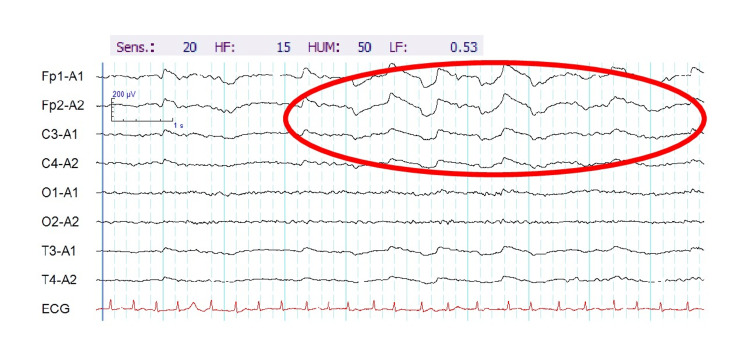
Post-hyperventilation electroencephalography (EEG) with eyes closed. The patient’s post-hyperventilation EEG results showed delta activity in the frontal lobe region (red oval).

The patient was diagnosed with an adjustment disorder caused by the stress of not receiving the treatment he wanted. Because of his psychiatric symptoms, rehabilitation and nursing care were difficult. On the morning of day 5, the patient presented with loss of consciousness with generalized convulsions. He also had left facial twitching, rightward conjugate deviation of the eyes, drooling, tachycardia with a heart rate of approximately 160 beats/min, and irregular respiration. At 10 minutes after onset, the symptoms improved without anticonvulsants or other drug therapy. Brain magnetic resonance imaging showed diffuse cortical atrophy to an age-appropriate degree. The possibilities of cerebral hemorrhage, cerebral infarction, and encephalitis were also considered. However, there were no findings suggestive of those conditions in the imaging conditions, including T2-star, diffusion-weighted, and fluid-attenuated inversion recovery (FLAIR) imaging. The patient’s unloaded electroencephalography (EEG) results also showed no obvious abnormalities (Figure 2). However, on post-hyperventilation, his EEG showed delta activity in the frontal lobe region (Figure 3). Blood tests were also performed on the patient, but there were no abnormalities in electrolytes, ammonia, blood sugar, or other factors that could contribute to convulsions. The patient was diagnosed with epileptic peri-ictal psychiatric symptoms.

Oral treatment with lacosamide 100 mg twice daily was started. The same seizure occurred once daily but disappeared on day 11 and did not recur thereafter. At approximately the same time, the patient’s irritability and mood swings also subsided. The patient and his wife and son were then involved in another review of the treatment plan. Increasing the dose of anagrelide did not control his abnormal platelet counts or coagulation system. Thus, rehabilitation progress involved a significant risk of hemorrhage and infarction. However, the patient and his family were aware of these risks and wanted active rehabilitation. Thus, the psychiatrist and hematologist respected the patient’s wishes, and the nurses and caseworkers actively supported the patient and his family during their visits. The patient became more active as rehabilitation progressed although he had persistent fever and fatigue. However, on day 31, he developed a hemorrhage from the central venous catheter insertion site in the right internal jugular vein. Attempts were made to stop the hemorrhage by re-stitching and compression, but the bleeding did not stop. On day 32, the patient died of hemorrhagic shock.

## Discussion

Thrombosis and prognosis in the high-risk ET group

Despite the good prognosis for ET patients, there are still high-risk groups for thrombosis and poor prognosis. As this is not a hematopathology report, detailed descriptions are omitted, but the abnormal white blood cell and platelet counts in this patient appeared to be caused by myelofibrosis caused by the JAK2 mutation [7-9]. Indeed, a risk classification incorporating JAK2 mutations was recently proposed [10]. Patients in the high-risk thrombosis group are typically treated with a combination of low-dose aspirin and cytoreductive therapy to prevent concomitant thrombosis [11,12]. Cytoreductive therapy includes hydroxyurea and anagrelide [12,13]. The present patient had good platelet count control by hydroxyurea after the initiation of ET treatment. However, an increase in the hydroxyurea dose was associated with adverse reactions, including lower limb ulceration, pneumonia, and heart failure. Therefore, re-administration of hydroxyurea was considered contraindicated. Hence, the only option for cytoreductive therapy was anagrelide. Unfortunately, this drug was unsuccessful and it was difficult to control his platelet count.

Psychiatric manifestations of epilepsy

Although there is no internationally recognized classification, it is practical to classify psychiatric symptoms according to their temporal relationship with epileptic seizures [14]. The psychiatric symptoms associated with epilepsy are mainly classified as peri-ictal (pre-ictal, ictal, postictal) and interictal [15]. The present patient showed psychiatric symptoms from three days prior to 11 days after the convulsive seizure, but not at any other time. Thus, the patient was diagnosed with epileptic peri-ictal psychiatric symptoms. Nevertheless, it should be included that, since this is a cutting-edge area of research, there were also different opinions among several experts on the diagnosis of this patient. In this case, the patient’s EEG findings did not directly lead to the diagnosis of epilepsy. However, the patient had two unprovoked seizures more than 24 hours apart and fit the diagnostic criteria of epilepsy by the International League Against Epilepsy (ILAE) [16]. Additionally, when abnormal waves are confirmed on EEG, it may not be possible to determine whether the patient has peri-ictal psychiatric symptoms. Therefore, in this case, a strict diagnosis was made on the 11th day, when the convulsions and mental symptoms disappeared. In the present case, the patient's EEG findings did not directly lead to the diagnosis of epilepsy, but the patient had two unprovoked seizures more than 24 hours apart, which met the diagnostic criteria for epilepsy according to the ILAE [16]. In addition, the presence or absence of seizure-phase psychiatric symptoms cannot always be determined when abnormal waves are identified on EEG, and in this case, the exact diagnosis was made on day 11, when the seizures and psychiatric symptoms had resolved.

Epilepsy and therapeutic agents in the elderly

Epilepsy is an important pitfall in the diagnosis of sudden psychiatric symptoms in the elderly [3]. Peri-seizure psychiatric symptoms include psychic seizures, which are psychiatric symptoms of the seizure itself [17]. The focus is mainly on the temporal lobe, with the patient exhibiting ictal fear, strong aversion, and déjà vu [18]. In general, the incidence of seizure recurrence within five years after first-time unprovoked seizure cases is approximately 26%-35% [19,20]. Therefore, as a rule, antiepileptic drug treatment is not initiated for first unprovoked seizures. However, because the recurrence rate after the first seizure is higher in older (66%-90%) than in younger patients, treatment is often initiated after the first seizure [4]. Also, patients with prior brain injury, significant abnormal brain imaging findings, epileptiform abnormality on EEG, or nocturnal seizures have a higher risk of seizure recurrence. Therefore, treatment with antiepileptic drugs should be individualized after first unprovoked seizures. Additionally, the epileptic seizures in our patient significantly impaired his quality of life, which indicated the initiation of antiepileptic drugs immediately after the first seizure. Novel (or third-generation) antiepileptic drugs have been developed for use in epilepsy, including perampanel, lacosamide, levetiracetam, and brivaracetam. Perampanel, lacosamide, and levetiracetam are marketed in Japan, while brivaracetam is not yet available. Several studies have reviewed the efficacy of perampanel, lacosamide, and levetiracetam for epilepsy treatment in older patients. However, the most optimal drug remains unclear [5,6], and these drugs are currently used differently for each patient. In this case, lacosamide was selected because perampanel and levetiracetam are known to cause psychiatric symptoms.

Management of epilepsy for palliative care

The management of epilepsy in this patient led to a marked improvement in his quality of life during palliative care. Although the patient ultimately succumbed to complications from ET, his neurological symptoms were well-controlled, allowing for better communication with his family and more active participation in his care.

## Conclusions

In epilepsy care, both convulsive seizures and psychiatric symptoms are important therapeutic targets. Lacosamide, which can be administered orally or intravenously and has a low risk of side effects such as psychiatric symptoms, was chosen for the present patient. Symptom management of epilepsy using lacosamide might be useful in palliative care for patients with ET. There have been no reports of epileptic seizures associated with ET in palliative medicine. Therefore, this case report is novel and useful for psychiatrists engaged in palliative care medicine and liaison consultation psychiatry.

## References

[1] Arber DA, Orazi A, Hasserjian RP (2022). International Consensus Classification of Myeloid Neoplasms and Acute Leukemias: integrating morphologic, clinical, and genomic data. Blood.

[2] Franchini M, Mannucci PM (2020). Acquired von Willebrand syndrome: focused for hematologists. Haematologica.

[3] Ramsay RE, Macias FM, Rowan AJ (2007). Diagnosing of epilepsy in the elderly. Int Rev Neurobiol.

[4] de Biase S, Nilo A, Bernardini A, Gigli GL, Valente M, Merlino G (2019). Timing use of novel anti-epileptic drugs: is earlier better?. Expert Rev Neurother.

[5] Rohracher A, Kalss G, Kuchukhidze G (2021). New anti-seizure medication for elderly epilepsy patients - a critical narrative review. Expert Opin Pharmacother.

[6] Ruggeri M, Finazzi G, Tosetto A, Riva S, Rodeghiero F, Barbui T (1998). No treatment for low-risk thrombocythaemia: results from a prospective study. Br J Haematol.

[7] Wolanskyj AP, Schwager SM, McClure RF, Larson DR, Tefferi A (2006). Essential thrombocythemia beyond the first decade: life expectancy, long-term complication rates, and prognostic factors. Mayo Clin Proc.

[8] Passamonti F, Thiele J, Girodon F (2012). A prognostic model to predict survival in 867 World Health Organization-defined essential thrombocythemia at diagnosis: a study by the International Working Group on Myelofibrosis Research and Treatment. Blood.

[9] Barbui T, Vannucchi AM, Buxhofer-Ausch V (2015). Practice-relevant revision of IPSET-thrombosis based on 1019 patients with WHO-defined essential thrombocythemia. Blood Cancer J.

[10] Palandri F, Catani L, Testoni N (2009). Long-term follow-up of 386 consecutive patients with essential thrombocythemia: safety of cytoreductive therapy. Am J Hematol.

[11] Harrison CN, Campbell PJ, Buck G (2005). Hydroxyurea compared with anagrelide in high-risk essential thrombocythemia. N Engl J Med.

[12] Matsuura M, Adachi N, Oana Y, Okubo Y, Hara T, Onuma T (2000). Proposal for a new five-axis classification scheme for psychoses of epilepsy. Epilepsy Behav.

[13] Gisslinger H, Gotic M, Holowiecki J (2013). Anagrelide compared with hydroxyurea in WHO-classified essential thrombocythemia: the ANAHYDRET Study, a randomized controlled trial. Blood.

[14] Shavit L, Grenader T, Galperin I (2012). Nonconvulsive status epilepticus in elderly a possible diagnostic pitfall. Eur J Intern Med.

[15] (2024). Clinical practice guidelines for epilepsy. https://www.neurology-jp.org/guidelinem/epilepsy/documents/guideline2018.pdf.

[16] Fisher RS, Acevedo C, Arzimanoglou A (2014). ILAE official report: a practical clinical definition of epilepsy. Epilepsia.

[17] Yamada N (2011). Psychiatric comorbidity in epilepsy [Article in Japanese]. Japan J Gen Hosp Psychiatry.

[18] Fisher RS, Cross JH, French JA (2017). Operational classification of seizure types by the International League Against Epilepsy: Position Paper of the ILAE Commission for Classification and Terminology. Epilepsia.

[19] Hauser WA, Rich SS, Lee JR, Annegers JF, Anderson VE (1998). Risk of recurrent seizures after two unprovoked seizures. N Engl J Med.

[20] Hauser WA, Rich SS, Annegers JF, Anderson VE (1990). Seizure recurrence after a 1st unprovoked seizure: an extended follow-up. Neurology.

